# Different Lipid Parameters in Predicting Clinical Outcomes in Chinese Statin-Naïve Patients After Coronary Stent Implantation

**DOI:** 10.3389/fcvm.2021.638663

**Published:** 2021-03-16

**Authors:** Li Zeng, Ziwei Ye, Ying Li, Yiling Zhou, Qingyang Shi, Tao Hu, Minghuan Fu, Caojie Wu, Haoming Tian, Sheyu Li

**Affiliations:** ^1^Department of Endocrinology and Metabolism, West China Hospital, Sichuan University, Chengdu, China; ^2^Department of Geriatrics, Sichuan Academy of Medical Sciences and Sichuan Provincial People's Hospital, Chengdu, China; ^3^Department of Guideline and Rapid Recommendation, Cochrane China Center, MAGIC China Center, Chinese Evidence-Based Medicine Center, West China Hospital, Sichuan University, Chengdu, China

**Keywords:** lipid profile, percutaneous coronary intervention, coronary artery disease, predictive factor, low-density lipoprotein

## Abstract

**Background:** Low-density lipoprotein cholesterol (LDL-C) is a critical surrogate outcome for cardiovascular disease (CVD). Recent observational studies identified different predictive lipid parameters, but these have not been fully validated in the Chinese population. This study aimed to compare the predictive value of lipid parameters for cardiovascular outcomes in Chinese statin-naïve patients who underwent percutaneous coronary intervention (PCI).

**Methods:** We retrospectively recruited statin-naïve patients who underwent PCI for stable angina and acute coronary syndrome at Sichuan Provincial People's Hospital between 1 January 2016 and 31 December 2017. A follow-up was conducted via outpatient visits or telephone. We divided patients into three groups based on lipid parameter tertiles. We calculated the hazard ratios (HRs) of the highest and lowest tertiles for major adverse cardiovascular events (MACEs) using multivariate Cox proportional hazards regression. We compared the association strength of lipid parameters with MACEs using the HR of non-LDL-C lipid parameters relative to LDL-C.

**Results:** Among 445 included patients, the highest LDL-C, LDL-C/high-density lipoprotein cholesterol (HDL-C), atherosclerosis index, and non-HDL-C level tertiles were associated with an average increase of 165% (HR 2.65, confidence interval [CI] 1.26 to 5.61; *P* = 0.01), 324% (HR 4.24, CI 1.89 to 9.52; *P* < 0.001), 152% (HR 2.52, CI 1.22 to 5.22; *P* = 0.01), and 125% (HR 2.25, CI 1.09 to 4.64; *P* = 0.01) in the hazard of composite CVD, respectively. Lipoprotein (a) levels did not show a significant association with the endpoints. Except for LDL-C/HDL-C, different lipid parameter HR ratios were <1.0; none were statistically significant.

**Conclusion:** Compared with non-LDL-C lipid parameters, LDL-C acts better predictive value for cardiovascular outcomes in general Chinese statin-naïve post-PCI patients.

## Introduction

Cardiovascular disease (CVD) is a leading cause of death globally (1). Post-percutaneous coronary intervention (PCI) patients represent a special CVD population (2), as they require secondary prevention for coronary heart disease (3). Predicting further CVD risk in such populations is critically relevant for clinical decision making, and novel factors that are potentially associated with CVD risk are being investigated (4). Lipid profiles are the most critical factors for CVD prevention, and low-density lipoprotein cholesterol (LDL-C) is a key risk factor for CVD (5, 6). Recent studies suggest that different lipid parameters, including lipoprotein (a), are strong predictors of CVD and perform better in patients receiving statin therapy than others (7–9). Atherogenic index of plasma is easy and reflects the small dense LDL, which is feasible for prevention and control of cardiovascular diseases in a community population and a strong marker for CAD in postmenopausal women (10). However, no studies have directly compared the predictive value of different lipid parameters in the Chinese population. Most previous studies enrolled patients taking various doses of statins and patients not taking statins. Such mixed populations lead to complicated confounding that cannot be fully adjusted for using statistics. In the current study, we recruited patients who underwent PCI and who had not previously received statins or other lipid-lowering treatments. This study aimed to investigate the value of recent different lipid parameters to predict cardiovascular outcomes.

## Materials and Methods

### Study Population

This study retrospectively recruited statin-naïve patients underwent PCI for stable angina, and acute coronary syndrome at Sichuan Provincial People's Hospital, Chengdu, China, between 1 January 2016 and 31 December 2017. We only included patients who had not used statins or other lipid-lowering drugs within 3 months before admission, and excluded patients with incomplete baseline or contact information for follow-up, or died not due to cardiovascular cause during initial hospitalization. Follow-up data were collected until 30 May 2020. The study was approved by the Biomedical Ethics Committee of Sichuan Provincial People's Hospital. Informed consent was obtained from all patients.

### Demographic and Clinical Characteristics

We conducted a chart review to collect data including the demographic information, comorbidities, body weight, height, and blood pressure data. Body mass index (BMI) was calculated as follows: body weight (kg) / height square (m^2^). We identified patients with hypertension as those who took antihypertensive drugs before admission or who were diagnosed with hypertension during hospitalization and patients with diabetes mellitus as those who took anti-diabetic agents or who were diagnosed with diabetes mellitus at hospital.

### Laboratory Examination

Laboratory data were collected, including total cholesterol, triglyceride, high-density lipoprotein cholesterol (HDL-C), LDL-C, lipoprotein (a), apolipoprotein B, apolipoprotein A-I, alanine aminotransferase, aspartate aminotransferase, uric acid, homocysteine, thyroid-stimulating hormone, total triiodothyronine, free triiodothyronine, total thyroxine, free thyroxine, and serum creatinine. The estimated glomerular filtration rate was calculated using the Chronic Kidney Disease Epidemiology Collaboration formula (11), and chronic kidney disease was defined as an estimated glomerular filtration rate of <60 mL/min/1.73 m^2^. Non-HDL-C was calculated as follows: total cholesterol—HDL-C (12). Atherosclerosis index was calculated as follows: (total cholesterol—HDL-C)/HDL-C (10). The atherogenic index of plasma was defined as the base-10 logarithm of the ratio of the concentration of triglyceride to HDL-C (10). The lipoprotein combine index was defined as the ratio of the product of total cholesterol, triglyceride, and LDL-C to HDL-C (13).

### Angiographic Data

Angiographic data were obtained from the image reporting system. Coronary severity was assessed using the Gensini score system (14). Two experienced interventional cardiologists independently calculated the Gensini score for each patient following a standardized approach and solved disagreement by discussion and re-calculation.

Triple-vessel disease was defined as angiographic stenosis of ≥50% in all three main epicardial coronary arteries, including the left anterior descending, left circumflex, and right coronary arteries.

### Follow-Up and Endpoints

The investigators followed up with patients by telephone calls, home visits, or chart reviews at Sichuan Provincial People's Hospital. All data collectors were trained for data entry and extraction.

We defined major adverse cardiovascular events (MACEs) as cardiovascular death, stroke, myocardial infarction, or ischemia-driven revascularization. Only episodes of PCI and coronary artery bypass surgery for ischemic symptoms were considered as endpoints.

The follow-up duration was determined from the discharge date to the first occurrence of an endpoint. If no endpoints occurred, data were collected until the last visit. Patients without endpoints were censored by the end of the last follow-up visit.

### Statistical Analysis

All statistical analyses were performed using R (R Pack 3.6.1) (15). We measured the normality of distribution using the Kolmogorov–Smirnov test. Normally distributed continuous variables were presented as mean ± standard deviation. Non-normally distributed continuous variables were presented as median and interquartile range. Categorical variables were presented as frequencies (percentages). We grouped patients into three groups based on the tertiles of each baseline lipid parameter. We compared the baseline characteristics of participants with and without MACE using student *t*-test, Mann-Whitney *U* test, or Chi-square test. We compared the risk of the first MACE in the highest tertile of each lipid parameter to its lowest tertile using multivariate Cox proportional hazards models with adjusted variables, baseline age, sex, body mass index, diabetes mellitus, hypertension, and Gensini score. We reported the hazard ratio (HR) and 95% confidence interval (CI). We used the ratio of HR (RHR) to measure the relative magnitude of the paired HR to find the largest effect size of different HRs. CIs of RHRs were calculated using the bootstrapping method. We set 5000 resamplings to generate bootstrap samples and calculated each as a bootstrap RHR. We chose the 2.5th and 97.5th percentiles as the 95% CI limits (16). A two-sided *P* value of < 0.05 was considered statistically significant.

## Results

### Patients

**Figure 1:**
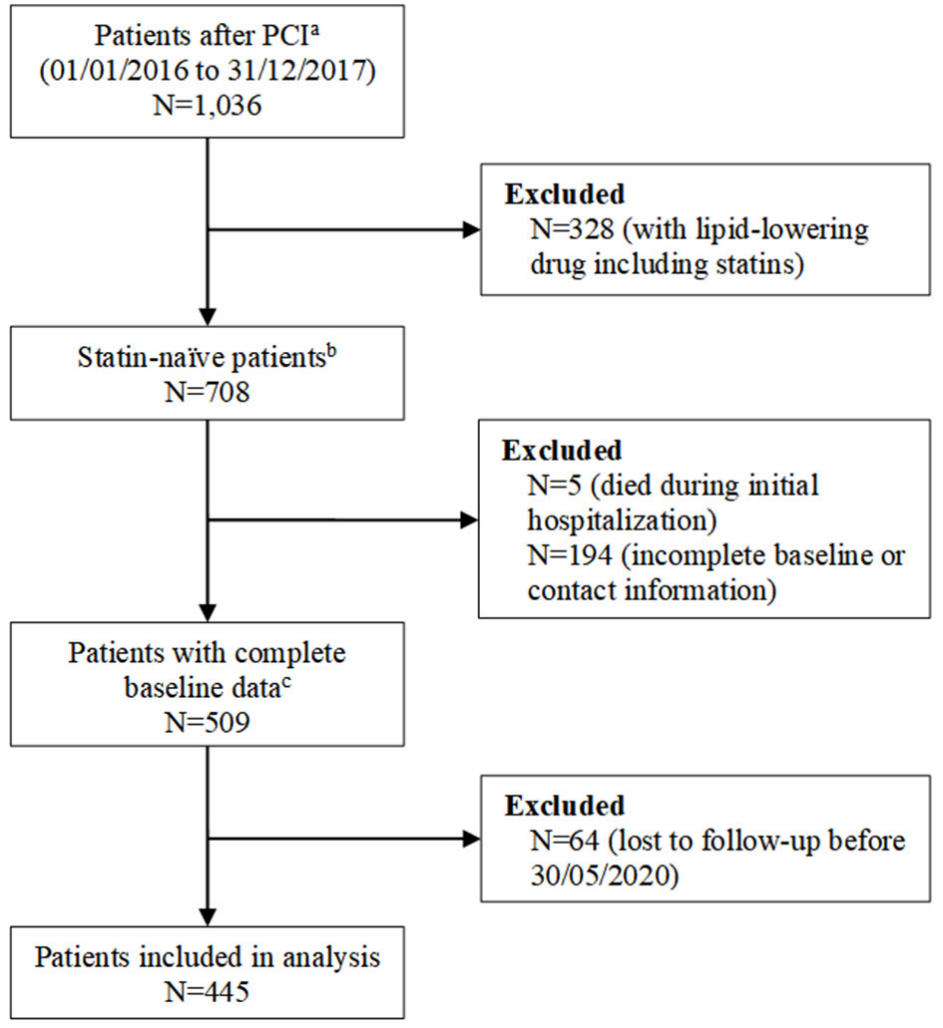
Study flowchart. ^**a**^Patients who were admitted for percutaneous coronary intervention (PCI) for stable angina and acute coronary syndrome at Sichuan Provincial People's Hospital, Chengdu, China, between 1 January 2016 and 31 December 2017. ^**b**^Patients did not use statin or other lipid-lowering drugs within 3 months before admission. ^**c**^Baseline data includes age, sex, prior medical history, comorbidities, serum creatinine, alanine aminotransferase, aspartate aminotransferase, uric acid, total cholesterol, triglyceride, high-density lipoprotein cholesterol (HDL-C), low-density lipoprotein cholesterol (LDL-C), lipoprotein **(a)**, apolipoprotein B, apolipoprotein A-I, and angiographic data.

As shown in Figure 1, we analyzed 445 patients meeting the eligibility criteria among 1,036 candidates who underwent PCI during the study period. Table 1 shows the baseline characteristics of patients. The median age of patients was 65.0 years (P25 to P75, 59.0 to 71.0 years), 321 patients (72.1%) were male, 33.7% of patients had a triple-vessel disease, 19.8% had diabetes mellitus, and 53.3% had hypertension. When compared with patients without MACE, patients with MACE had a higher level of triglyceride, non-HDL-C, LDL-C/HDL-C, and atherogenic index of plasma, and a higher rate of diabetes. The differences of baseline characteristics of patients in different tertiles of LDL-C were shown in Supplementary Table 1.

**Table 1:** Baseline characteristics of participants.

**Characteristics**	**Total patients (*n* = 445)**	**Patients without MACE (*n* = 385)**	**Patients with MACE (*n* = 60)**	***P* value**
Age (years)	65.0 (59.0, 71.0)	65.0 (59.0, 71.0)	64.5 (55.3, 72.0)	0.59
Male, *n* (%)	321 (72.1)	280 (72.7)	41 (68.3)	0.54
BMI (kg/m^2^)	24.1 ± 3.3	24.3 ± 3.2	24.9 ± 3.7	0.19
Diabetes, *n* (%)	88 (19.8)	67 (17.4)	21 (35.0)	0.003
Hypertension, *n* (%)	237 (53.3)	201 (52.2)	36 (60.0)	0.27
Gensini score	37.0 (20.0, 59.5)	34.0 (20.0, 58.0)	48.5 (28.0, 80.0)	<0.001
TVD, *n* (%)	150 (33.7)	125 (32.5)	25 (41.7)	0.19
Stent before, *n* (%)	48 (10.8)	41 (10.7)	7 (11.7)	0.82
eGFR (ml/min/1.73 m^2^)	89.9 (77.4, 98.8)	89.9 (78.0, 58.0)	88.6 (70.5, 100.2)	0.10
Cr (μmol/L)	72.6 (61.3, 86.8)	72.3 (61.2, 86.2)	76.4 (62.6, 89.5)	0.13
Ur (μmol/L)	356.0 (297.0, 432.0)	354.0 (296.5, 427.0)	367.0 (306.5, 443.8)	0.37
AST (IU/L)	34.0 (26.0, 62.0)	34.0 (26.0, 63.5)	35.0 (24.0, 50.8)	0.40
ALT (IU/L)	27.0 (19.0, 43.0)	27.0 (19.0, 43.0)	27.5 (18.3, 46.0)	0.91
TSH (mIU/L)	1.5 (1.0, 2.6)	1.6 (1.0, 2.6)	1.5 (0.8. 2.3)	0.62
TT3 (nmol/L)	1.5 ± 0.3	1.5 ± 0.3	1.4 ± 0.3	0.23
TT4 (nmol/L)	82.1 ± 17.2	82.1 ± 17.0	81.9 ± 18.3	0.93
FT3 (pmol/L)	4.0 ± 0.7	4.0 ± 0.7	3.9 ± 0.8	0.28
FT4 (pmol/L)	13.2 ± 1.9	13.16 ± 1.8	13.4 ± 2.3	0.38
Homocysteine (μmol/L)	17.4 ± 6.4	17.4 ± 6.6	17.5 ± 5.1	0.92
Total cholesterol (mmol/L)	5.1 (4.5, 5.7)	5.0 (4.4, 5.6)	5.3 (4.5, 6.1)	0.06
Triglyceride (mmol/L)	1.8 (1.3, 2.7)	1.8 (1.3, 2.6)	1.96 (1.4, 3.9)	0.002
LDL-C (mmol/L)	3.2 (2.7, 3.7)	3.2 (2.7, 3.7)	3.4 (3.0, 3.8)	0.04
HDL-C (mmol/L)	1.1 ± 0.3	1.1 (0.9, 1.3)	1.0 (0.9, 1.2)	0.06
Apolipoprotein B (g/L)	1.0 (0.8, 1.2)	1.0 (0.8, 1.2)	1.0 (0.9, 1.3)	0.43
Apolipoprotein A-I (g/L)	1.3 (1.1, 1.4)	1.3 (1.1, 1.4)	1.2 (1.1, 1.3)	0.13
Non-HDL-C (mmol/L)	3.9 (3.4, 4.5)	3.9 (3.4, 4.5)	4.2 (3.6, 5.0)	0.02
Lp(a) (mg/dl)	196.0 (84.0, 472.5)	193.0 (84.0, 495.0)	200.0 (72.8, 407.0)	0.54
LDL-C/HDL-C	3.0 (2.4, 3.6)	2.9 (2.3, 3.5)	3.4 (2.7, 4.2)	0.004
Atherogenic index of plasma	0.2 ± 0.3	0.2 ± 0.3	0.4 ± 0.4	0.001
Atherosclerosis index	3.7 (3.0, 4.5)	3.6 (2.9, 4.4)	4.2 (3.3, 5.6)	0.006
Lipoprotein combine index	28.0 (17.0, 48.0)	27.7 (15.7, 44.9)	34.9 (20.0, 96.2)	0.003
Apolipoprotein B/apolipoprotein A-1	0.8 (0.6, 1.0)	0.8 (0.6, 1.0)	0.9 (0.7, 1.0)	0.17

*BMI, body mass index; TVD, triple-vessel disease; eGFR, estimated glomerular filtration rate; Cr, creatinine; Ur, uric acid; AST, aspartate aminotransferase; ALT, alanine aminotransferase; TSH, thyroid-stimulating hormone; TT3, total triiodothyronine; FT3, free triiodothyronine; TT4, total thyroxine; FT4, free thyroxine; LDL-C, low-density lipoprotein cholesterol; HDL-C, high-density lipoprotein cholesterol; Lp(a), lipoprotein (a); MACE, major adverse cardiovascular events*.

### Follow-Up and Endpoints

The date of the last follow-up contact with a patient was 30 May 2020. The median duration of follow-up was 36 months (interquartile range 27 to 41 months) with an incidence of 4.9 cardiovascular events per 100 person-years. MACEs occurred in 60 patients (13.5%).

**Figure 2:**
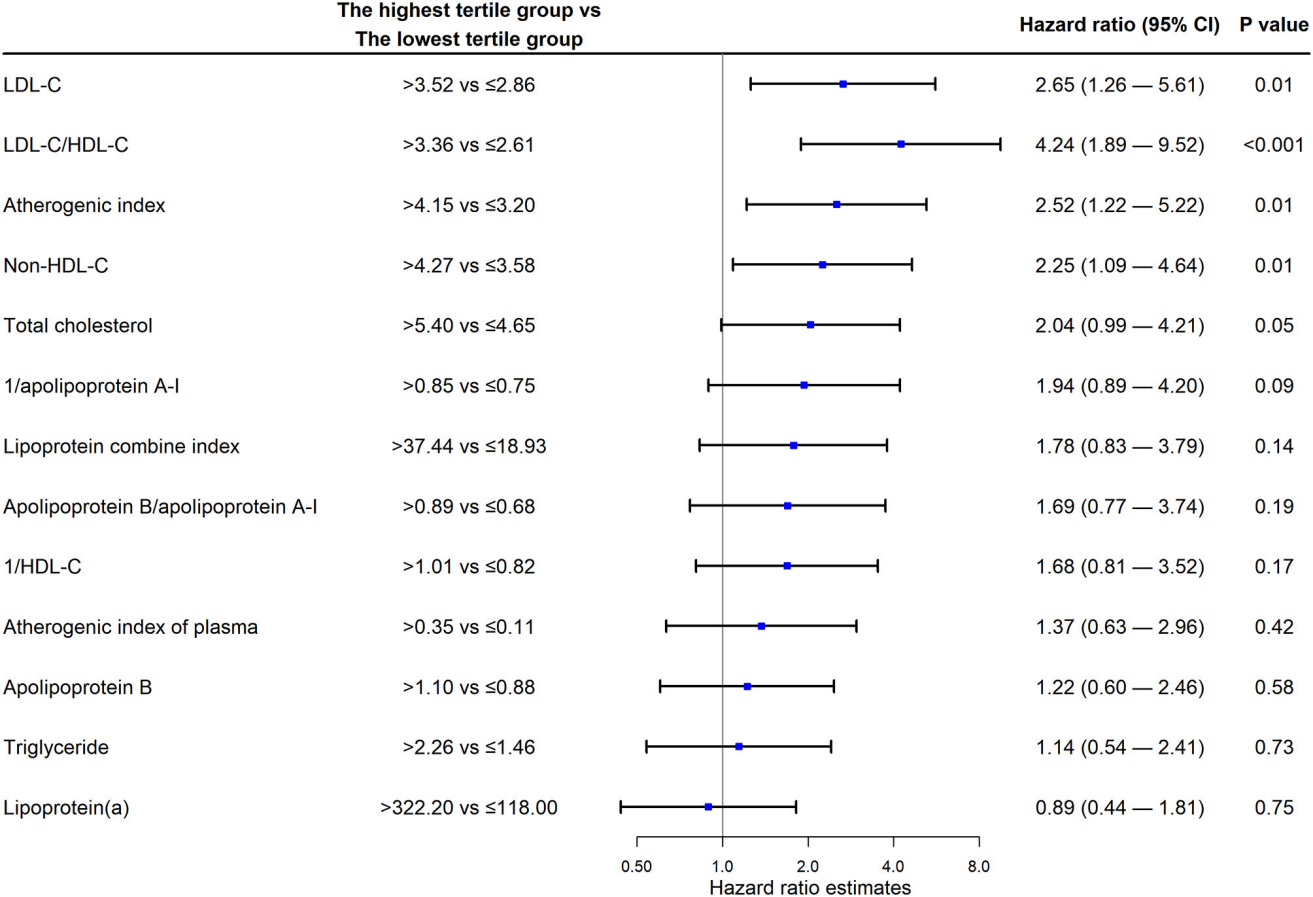
Cox regression analysis. LDL-C, low-density lipoprotein cholesterol; HDL-C, high-density lipoprotein cholesterol; CI, confidence interval.

As shown in Figure 2, the multivariate Cox proportional hazards model showed that the HR of the highest LDL-C group compared with the lowest LDL-C group was the highest (HR 2.65; 95% CI 1.26 to 5.61; *P* = 0.01). Non-HDL-C level (HR 2.25; 95% CI 1.09 to 4.64; *P* = 0.01), atherosclerosis index (HR 2.52; 95% CI 1.22 to 5.22; *P* = 0.01), and LDL-C/HDL-C ratio (HR 4.24; 95% CI 1.89 to 9.52; *P* < 0.001) were independently associated with an increased risk of MACEs (Figure 2). Lipoprotein (a) levels did not show an obvious association with the endpoint (HR 0.89; 95% CI 0.44 to 1.81). The HRs for the middle tertile of those parameters compared with their lowest tertile were not statistically significant (Supplementary Figure 1).

**Figure 3:**
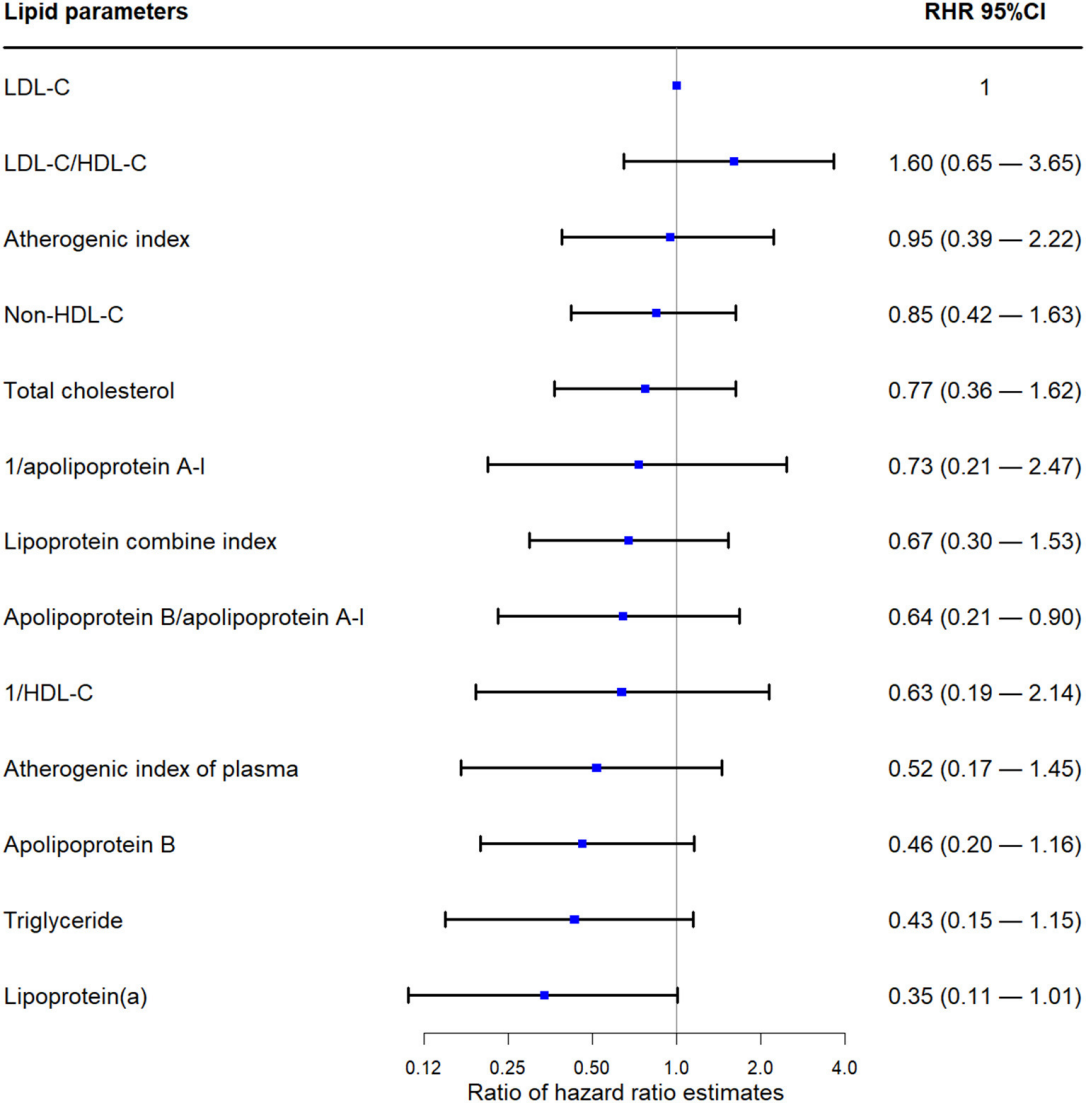
Ratio hazard ratio of different lipid parameters. LDL-C, low-density lipoprotein cholesterol; HDL-C, high-density lipoprotein cholesterol; CI, confidence interval.

As shown in Figure 3, all RHRs of lipid parameters (except for LDL-C/HDL-C) were <1.00. The LDL-C/HDL-C ratio did not show significantly better performance compared with LDL-C (RHR 1.60; 95% CI 0.65 to 3.65).

## Discussion

Our study shows that baseline LDL-C level is independently associated with MACEs in statin-naïve patients after PCI. None of the different lipid parameters performed better than LDL-C in the association with cardiovascular outcomes by a tertile grouping. This is the first study to compare different lipid parameters in Chinese statin-naïve patients after PCI.

Recent longitudinal studies suggest a strong prediction effect of lipoprotein (a) in patients at risk of atherosclerotic CVD (17). European guidelines in 2019 recommended lipoprotein (a) in patients with a family history of premature CVD and for reclassification in moderate-to-high-risk patients (12). However, our study did not show an association between baseline lipoprotein (a) level tertile and cardiovascular events in Chinese statin-naïve patients after PCI. Our findings are consistent with a previous study in China (18). This ethnic difference may be related to the absolute concentration and isoform sizes of lipoprotein (a), as Chinese people were found to have the lowest lipoprotein (a) concentration (median = 7.8 mg/dL) and the largest isoform size (median = 28) among seven major ethnic groups (19). This suggests that we need to be aware of the difference across ethnicity and the difference between randomized trials and real-world population (20). It may not be reasonable to measure lipoprotein (a) routinely in all patients undergo PCI. Further study is warranted to investigate the target population needing lipoprotein (a) measurement.

In our study, none of the lipid parameters performed better than LDL-C level, except for the LDL-C/HDL-C, despite some of those parameters being recommended in Western guidelines (21, 22). This suggests that LDL-C remains the best predictive factor for cardiovascular events in the secondary prevention of CVD. This finding is in line with LDL-C-targeting lipid-lowering trials (23). LDL-C/HDL-C is a good predictor of cardiovascular events and atherosclerosis (24, 25). However, few lipid-lowering drugs elevate HDL-C with cardiovascular benefits (26). Neither HDL-C nor LDL-C/HDL-C is a better treatment target for cardiovascular prevention than LDL-C, which is the pharmaceutical target of major lipid-lowering drugs that reduce cardiovascular events, including statins, ezetimibe, and PCSK9 inhibitors (27–29).

There are three major limitations in our study. First, this is a single-center study with a limited sample size and event rate. Further study is warranted at other cardiovascular centers to validate the results. Second, we did not measure the subtypes of LDL-C, very low-density lipoprotein cholesterol, or lipoprotein-associated phospholipase A2, which are not routinely measured in clinical practice. Finally, we could not assess the prevalence of patients with familial hyperlipidemia, which could not be assessed based on the current data.

## Conclusions

In summary, our findings indicate that LDL-C remains the best lipid parameter associated with cardiovascular events in general Chinese statin-naïve patients after PCI. External validation of our study in a larger population is warranted.

## Data Availability Statement

The raw data supporting the conclusions of this article will be made available by the authors, without undue reservation.

## Ethics Statement

The studies involving human participants were reviewed and approved by Biomedical Ethics Committee of Sichuan Provincial People's Hospital. Written informed consent for participation was not required for this study in accordance with the national legislation and the institutional requirements.

## Author Contributions

LZ, HT, and SL conceived the study. YL, MF, TH, and CW followed up the patients and was responsible for the data collection. LZ, YZ, and QS analyzed the data. QS and SL supervised the statistical analysis. LZ, ZY, HT, and SL interpreted the results. LZ, YZ, and SL drafted the manuscript. All authors read and approved the final version of this manuscript. LZ, HT and SL were responsible for the study design, data collection, and data analysis and manuscript writing.

## Conflict of Interest

The authors declare that the research was conducted in the absence of any commercial or financial relationships that could be construed as a potential conflict of interest.

## Supplementary Material

Supplementary Figure 1 Cox regression analysis comparing T2 vs. T1. LDL-C, low-density lipoprotein cholesterol; HDL-C, high-density lipoprotein cholesterol; CI, confidence interval; T1, the lowest tertile; T2, the middle tertile.

Supplementary Table 1 Baseline characteristics of patients in different tertiles of low-density lipoprotein cholesterol. MACE, major adverse cardiovascular events; BMI, body mass index; TVD, triple-vessel disease; eGFR, estimated glomerular filtration rate; Cr, creatinine; Ur, uric acid; AST, aspartate aminotransferase; ALT, alanine aminotransferase; TSH, thyroid-stimulating hormone; TT3, total triiodothyronine; FT3, free triiodothyronine; TT4, total thyroxine; FT4, free thyroxine; LDL-C, low-density lipoprotein cholesterol; HDL-C, high-density lipoprotein cholesterol; LP(a), lipoprotein (a); T1, the lowest tertile; T2, the middle tertile; T3, the highest tertile.
