# Supplementary figures and images for: Different Lipid Parameters in Predicting Clinical Outcomes in Chinese Statin-Naïve Patients After Coronary Stent Implantation
Source: Front Cardiovasc Med. 2021 Mar 16;8:638663. doi: 10.3389/fcvm.2021.638663 (PMC8007761; doi:10.3389/fcvm.2021.638663)

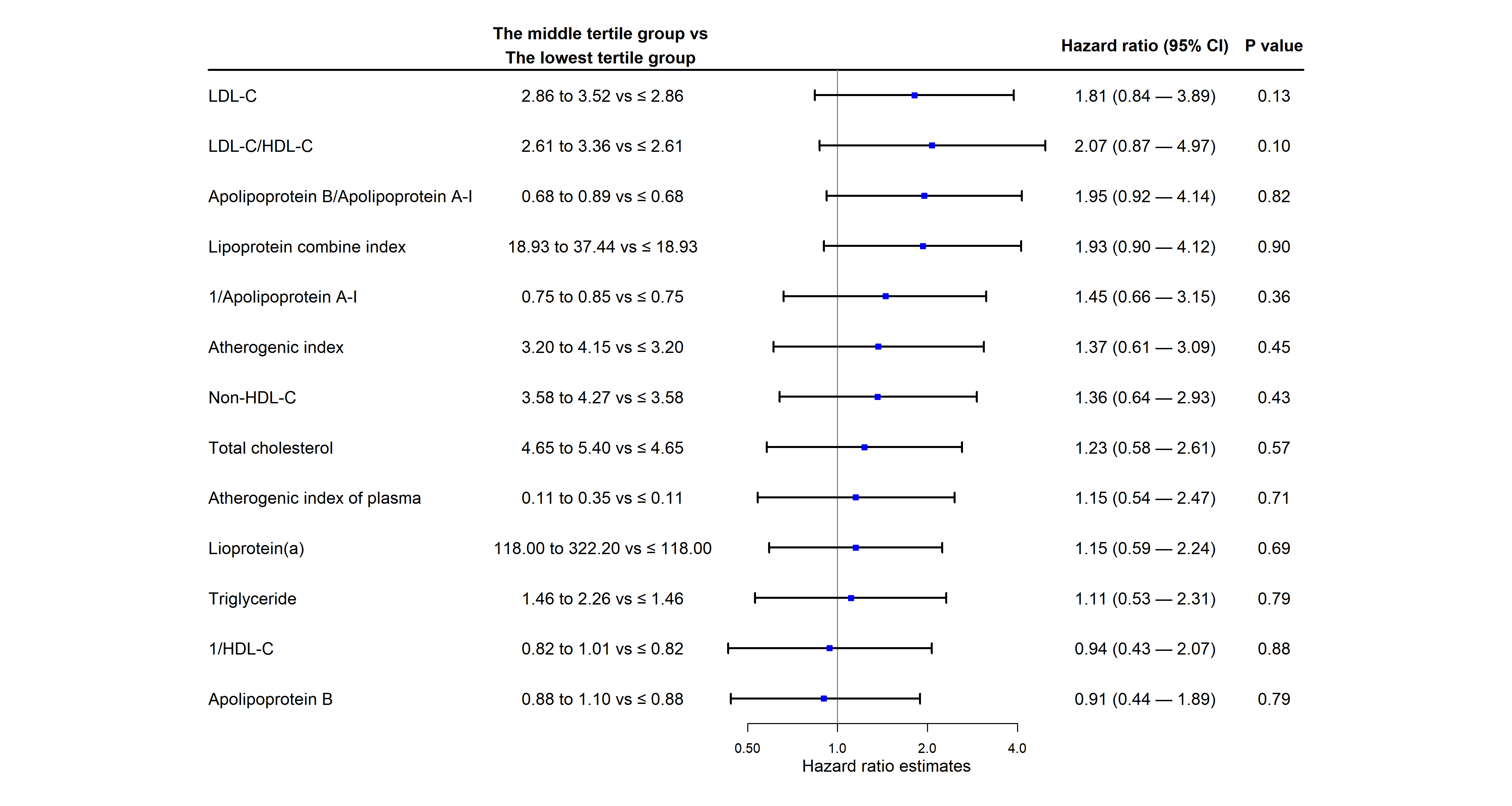

Supplement: Supplementary Figure 1 — Cox regression analysis comparing T2 vs. T1. LDL-C, low-density lipoprotein cholesterol; HDL-C, high-density lipoprotein cholesterol; CI, confidence interval; T1, the lowest tertile; T2, the middle tertile. [file Image_1.PNG]
