# Supplementary material for: Different Lipid Parameters in Predicting Clinical Outcomes in Chinese Statin-Naïve Patients After Coronary Stent Implantation
Source: Front Cardiovasc Med. 2021 Mar 16;8:638663. doi: 10.3389/fcvm.2021.638663 (PMC8007761; doi:10.3389/fcvm.2021.638663)
Supplement: Supplementary Table 1 — Baseline characteristics of patients in different tertiles of low-density lipoprotein cholesterol. MACE, major adverse cardiovascular events; BMI, body mass index; TVD, triple-vessel disease; eGFR, estimated glomerular filtration rate; Cr, creatinine; Ur, uric acid; AST, aspartate aminotransferase; ALT, alanine aminotransferase; TSH, thyroid-stimulating hormone; TT3, total triiodothyronine; FT3, free triiodothyronine; TT4, total thyroxine; FT4, free thyroxine; LDL-C, low-density lipoprotein cholesterol; HDL-C, high-density lipoprotein cholesterol; LP(a), lipoprotein (a); T1, the lowest tertile; T2, the middle tertile; T3, the highest tertile. [file Table_1.DOC]

**Supplementary materials**

**Supplementary Table 1.** Baseline characteristics of patients in different tertiles of low-density lipoprotein cholesterol

| **Characteristics (n = 445)** | **T1 (n = 147)** | **T2 (n = 147)** | **T3 (n = 151)** | **P value** |
| --- | --- | --- | --- | --- |
| **Age (years)** | 66.0 (60.0, 71.0) | 65.0 (56.0, 71.0) | 64.0 (58.0, 72.0) | 0.40 |
| **Male, n (%)** | 110 (74.8) | 111 (75.5) | 100 (66.2) | 0.14 |
| **BMI (kg/m^2^)** | 24.5 ± 3.3 | 24.1 ± 3.2 | 24.6 ± 3.2 | 0.40 |
| **Diabetes, n (%)** | 25 (16.6) | 22 (15.0） | 41 (27.9) | 0.01 |
| **Hypertension, n (%)** | 82 (34.6) | 73 (49.7) | 82 (54.3) | 0.55 |
| **Gensini score** | 35.0 (20.0, 52.0) | 34.0 (20.0, 64.0) | 42.0 (23.0, 62.0) | 0.13 |
| **TVD, n (%)** | 43 (29.3) | 47 (32.0) | 60 (39.7) | 0.14 |
| **Stent before, n (%)** | 19 (12.9) | 17 (11.6) | 12 (7.9) | 0.35 |
| **eGFR (ml/min/1.73m^2^)** | 88.1 (69.4, 97.5) | 91.0 (75.9, 99.0) | 90.6 (80.7, 99.5) | 0.10 |
| **Cr (μmol/L)** | 76.5 (62.2, 92.0) | 74.3 (63.9, 99.3) | 68.1 (59.4, 81.8) | 0.01 |
| **Ur (μmol/L)** | 356.0 (298.0, 438.0) | 364.0 (302.0, 434.0) | 349.0 (288.0, 423.0) | 0.70 |
| **AST (IU/L)** | 35.0 (26.0, 71.0) | 35.0 (27.0, 62.0) | 33.0 (25.0, 54.0) | 0.50 |
| **ALT (IU/L)** | 28.0 (20.0, 48.0) | 28.0 (18.0, 45.0) | 27.0 (19.0, 42.0) | 0.30 |
| **TSH (mIU/L)** | 1.5 (1.0, 2.5) | 1.5 (0.9, 2.6) | 1.6 (1.1, 2.7) | 0.50 |
| **TT3 (nmol/L)** | 1.5 ± 0.3 | 1.4 ± 0.3 | 1.5 ± 0.3 | 0.30 |
| **TT4 (nmol/L)** | 86.2 ± 17.3 | 79.1 ± 14.9 | 82.1 ± 18.7 | 0.01 |
| **FT3 (pmol/L)** | 4.0 ± 0.6 | 4.0 ± 0.8 | 4.0 ± 0.7 | 0.96 |
| **FT4 (pmol/L)** | 13.3 ± 1.9 | 13.0 ± 1.7 | 13.2 ± 2.1 | 0.50 |
| [**Homocysteine (μmol/L)**](javascript:;) | 17.0 (13.0, 20.5) | 16.6 (12.5, 22.2) | 17.0 (13.4, 19.7) | 0.80 |
| **Total cholesterol (mmol/L)** | 4.3 (4.0, 4.7) | 5.0 (4.7, 5.3) | 5.8 (5.5, 6.4) | <0.001 |
| **Triglyceride (mmol/L)** | 1.8 (1.3, 2.7) | 1.8 (1.3, 2.5) | 1.9 (1.4, 2.8) | 0.30 |
| **HDL-C (mmol/L)** | 1.1 (0.9, 1.3) | 1.1 (0.9, 1.3) | 1.1 (0.9, 1.3) | 0.30 |
| **Apolipoprotein B (g/L)** | 0.8 (0.7, 0.9) | 0.9 (0.9, 1.1) | 1.2 (1.1, 1.3) | <0.001 |
| **Apolipoprotein A-I (g/L)** | 1.2 (1.1, 1.4) | 1.3 (1.1, 1.4) | 1.2 (1.1, 1.3) | 0.80 |
| **Non-HDL-C (mmol/L)** | 3.2 (3.0, 3.5) | 3.9 (3.6, 4.1) | 4.7 (4.4, 5.2) | <0.001 |
| **Lp(a) (mg/dl)** | 141.0 (67.5, 337.0) | 201.0 (100.0, 432.0) | 261.0 (121.0, 624.5) | <0.001 |
| **LDL-C/HDL-C** | 2.3 (1.9, 2.9) | 3.0 (2.5, 3.5) | 3.5 (3.1, 4.2) | <0.001 |
| **Atherogenic index of plasma** | 0.2 ± 0.3 | 0.2 ± 0.3 | 0.3 ± 0.3 | <0.001 |
| **Atherosclerosis index** | 3.2 (2.4, 3.9) | 3.7 (3.0, 4.4) | 4.1 (3.6, 5.2) | <0.001 |
| **Lipoprotein combine index** | 17.5 (12.3, 30.11) | 26.7 (16.8. 43.6) | 41.0 (23.6, 72.7) | <0.001 |
| **Apolipoprotein B/apolipoprotein A-1** | 0.7 (0.5, 0.8) | 0.8 (0.6, 0.9) | 0.9 (0.8, 1.1) | <0.001 |

Abbreviation: MACE, major adverse cardiovascular events; BMI, body mass index; TVD, triple-vessel disease; eGFR, estimated glomerular filtration rate; Cr, creatinine; Ur, uric acid; AST, aspartate aminotransferase; ALT, alanine aminotransferase; TSH, thyroid-stimulating hormone; TT3, total triiodothyronine; FT3, free triiodothyronine; TT4, total thyroxine; FT4, free thyroxine; LDL-C, low-density lipoprotein cholesterol; HDL-C, high-density
